# Cross-Sectional Survey to Assess Dental Students' Perception of the Utilization of a Case Difficulty Assessment Form during Various Stages of Root Canal Therapy

**DOI:** 10.1155/2024/1217448

**Published:** 2024-05-29

**Authors:** Lubna E. Hamadah, Maha M. Algofaily, Ali T. Alaqla, Naif A. Alrubaiq, Ghaida A. Aljammaz

**Affiliations:** ^1^ Restorative and Prosthetic Dental Sciences College of Dentistry King Saud Bin Abdulaziz University for Health Sciences National Guard Health Affairs, Riyadh 11426, Saudi Arabia; ^2^ King Abdullah International Medical Research Center National Guard Health Affairs, Riyadh 11426, Saudi Arabia; ^3^ Restorative Dental Sciences College of Dentistry King Saud University, Riyadh 11481, Saudi Arabia; ^4^ King Abdulaziz Medical City National Guard Health Affairs, Riyadh 11426, Saudi Arabia

## Abstract

**Background:**

Several endodontic difficulty assessment forms are available to help dental students and general dentists estimate the difficulty of the case before initiating the endodontic treatment.

**Objectives:**

This study aimed to assess if the American Association of Endodontics (AAE) case difficulty assessment form affects the dental student's perception of the difficulty encountered while performing root canal treatment (RCT).

**Materials and Methods:**

This was a cross-sectional online survey. After obtaining IRB approval, an electronic questionnaire was sent to dental students enrolled in the 4th and 5th years at King Saud University (KSU) and King Saud bin Abdulaziz University for Health Sciences (KSAU-HS), Riyadh, Saudi Arabia. The questionnaire was divided into informed consent, demographic data, the use of the AAE case assessment form, RCT steps, diagnosis, local anesthesia administration, tooth isolation, and endodontic procedure.

**Results:**

A total of 195 dental students participated in the study. There were 101 (52%) females, and 139 (71%) were from KSAU-HS. A positive association was found between students who used the AAE assessment form and who did not with their ability to reach the correct diagnosis (*p*=0.005), tooth isolation (*p*=0.03), and endodontic procedure difficulty score (*p*=0.018).

**Conclusion:**

The use of the AAE form by dental students enabled them to differentiate between complicated and uncomplicated cases, thus lowering the difficulty encountered during RCT.

## 1. Introduction

An endodontic procedure requires knowledgeable and experienced dental practitioners to deliver high-quality root canal therapies [1]. It is challenging for dental students to confidently apply theoretical information and preclinical training to clinical practice. Recent studies have reported variable self-confidence levels of dental students during root canal treatment and a high prevalence of substandard root canal treatment outcomes performed by dental students [2, 3, 4]. Further information is needed to understand the reasons behind the low confidence level and dental students' difficulties while performing root canal treatment.

To enhance the confidence of dental students to perform root canal treatment, they must be adequately trained to select the suitable case to be treated by a general dentist and be able to refer the patient to an endodontist as needed. In 1992, Rosenberg and Goodis [5] had implemented a case selection form to help dental students at the University of California San Francisco (UCSF) in decision-making when to treat the case and when it should be referred to a specialist. Such a decision depends on the ability to assess oneself in addition to the skills and experience of the referring dentist [5].

Several case assessment tools are available nowadays to aid in difficulty assessment before the endodontic treatment. These can help in deciding whether it is better for the patient to be treated by a specialist. Various international organizations have developed different forms including endodontic case assessment form (ECAF), the Dutch Endodontic Treatment Index (DETI), and the American Association of Endodontics (AAE) case assessment form in this regard [1].

The AAE case difficulty assessment form is the most evaluated in the literature [6, 7]. It is a standardized document utilized to gather data and evaluate the amount of complexity associated with a particular case. The document encompasses potential risk factors that can complicate therapy and hurt outcomes. The criteria are categorized into patient considerations, diagnosis and treatment considerations, and other considerations. This form offers a standardized framework for general dentists to evaluate the complexity of a dental case impartially. The acquired information can be utilized to assess the patient on the complexity of the case and its future prognosis. It can also facilitate communication with a specialist if the general dentist deems it necessary to refer the patient due to its difficulty [8].

Dentists should reflect on their abilities to manage a case prior to commencing any endodontic procedure. If they lack the ability to do so, they should delegate the case to an expert who possesses the necessary skills to handle the case [9]. Therefore, it is imperative to evaluate a case before to commencing the treatment. The AAE developed the Endodontic Case Difficulty Assessment Form to provide practitioners with an objective means of assessing the level of difficulty of a case [8].

The use of the AAE case difficulty assessment form among the dental student and assessing their perception on the level of difficulty encountered while performing root canal treatment is fundamental in making informed judgments regarding the most beneficial treatment for the patient and better preparing students to practice endodontic procedures with a high level of confidence. All students who participated in this study underwent a comprehensive preclinical endodontic course in the second year as part of the curriculum. The student had to be competent in the practical test to pass the preclinical course successfully.

Based on the literature review, no studies reported an association between the AAE case assessment form and dental student confidence in performing nonsurgical root canal treatment. Thus, this study aimed to assess if using the AAE case assessment form affects the dental student's perception of the difficulty level encountered while performing each step during root canal treatment. Also, to identify the difficulties, the dental students face in achieving nonsurgical root canal treatment will be reported.

## 2. Materials and Methods

This cross-sectional study was conducted by distributing an e-questionnaire to dental students, both males and females, from the 4th and 5th years of the dental colleges from two universities in Riyadh, Saudi Arabia: College of Dentistry at King Saud bin Abdulaziz University for Health Sciences (KSAUHS) and College of Dentistry at King Saud University (KSU) during the academic year of 2022–2023 Questionnaire S1. The data were collected within a period of 2 months following the receipt of ethical approval from King Abdullah International Medical Research Centre (KAIMRC) IRB approval number 2657/22.

The questionnaire was developed by the research team. The content validity of this study was determined by quantitative analysis of expert judgment (Aiken's V). All the questions scored between 0.8 and 1 and thus were found to be valid. The questionnaire was pretested on a sample of 15 students to ensure that the questions were understood and answered appropriately.

A sample size of 385 was estimated based on 95% confidence level, 5% margin of error, and expected outcome response distribution of 50%. The selected students were enrolled in the last two clinical years of their dental school and had successfully passed the preclinical Endodontic course before their enrollment in the clinical course. Convenient sampling was used, and the questionnaire link was emailed to all the students enrolled in the 4th and 5th years of their dental school. Students who agreed to participate responded to the survey. The students affirmed their consent on the informed consent form prior to the commencement of the survey.

The questionnaire consisted of four main sections: informed consent, demographic data, the use of AAE endodontic case assessment form, root canal treatment steps, diagnosis, local anesthesia administration, isolation, and endodontic procedure. The demographic data section included questions regarding gender, university, and academic year level. The third section was a question related to the use of the AAE assessment form. The fourth section regarding the root canal treatment steps section was divided into four subsections: diagnosis, isolation, local anesthesia administration, and endodontic procedure. The respondent was instructed to rate each step using Likert scale from 1 to 5, with 1 being very easy and 5 being very difficult. The diagnosis subsection included 11 questions starting with gathering information about the history of the patient's chief complaint, performing cold test, electric pulp test, percussion, palpation, probing, bite test, establishing pulpal and periapical diagnosis, and analyzing diagnostic periapical and bitewing radiographs. The local anesthesia administration subsection section included questions related to performing different local anesthetic techniques which are local infiltration, regional block, and intrapulpal techniques. The isolation subsection included questions related to clamp selection, rubber dam application, and tooth buildup after caries excavation. Lastly, the endodontic procedure section included questions about each step of the root canal treatment process, starting from the access cavity preparation, achieving straight line access, glide path, using electronic apex locator to determine working length, recapitulation, using sodium hypochlorite irrigant, selection of master apical file, step back, placement and removal of intracanal medicament, master cone fit, obturation, and temporization to taking diagnostic radiographs.

Data were entered and analyzed using Statistical Package for the Social Sciences, SPSS version 23. Frequency and percentages were used to present the categorical variables. Minimum, maximum, mean, and standard deviation were used to present the numerical variables. Independent samples *t*-test was used to test for factors associated with the difficulty scores. The level of significance was set at 0.05 for all the statistical tests.

## 3. Results

A total of 195 dental student participated in the study with a response rate of 39.7%. Of these, 48.2% of the respondents were males, and 51.8% were females. The sociodemographic and academic profile of the participants are presented in Table 1.

**Figure 1:**
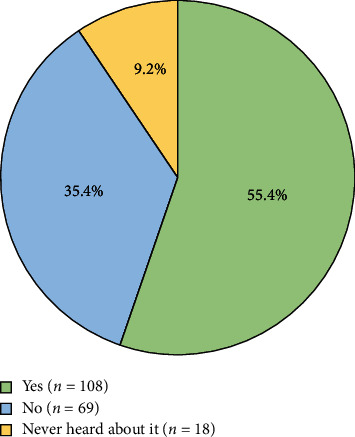
“Do you use the American Association of Endodontists (AAE) endodontic case assessment form?”

Among the participants, 108 (55.4%) reported using the American Association of Endodontists (AAE) endodontic case assessment form, 69 (35.4%) indicated they do not use it, and 18 (9.2%) mentioned they had never heard about it (Figure 1). The results of the study show a significant difference between students who used the AAE assessment form and who did not with their ability to reach the correct diagnosis (*p*=0.005), tooth isolation (*p*=0.03), and endodontic procedure difficulty score (*p*=0.018).

Students who reported using the AAE endodontic case assessment form reported a significantly lower diagnosis difficulty score compared to those who did not (*p*=0.005) (18.10 ± 4.83 vs. 20.10 ± 4.97) (Table 2). The academic level of KSAU students was significantly associated with diagnosis difficulty score (*p*=0.012), where it was observed that those in the 4th year had a significantly higher diagnosis difficulty score compared to those in the 5th year (19.73 ± 4.71 vs. 17.71 ± 4.53) (Table 2).

The use of the AAE endodontic case assessment form was significantly associated with isolation difficulty score, where it was observed that those who used it had a significantly lower isolation difficulty score (*p*=0.03) compared to those who did not (6.69 ± 2.13 vs. 7.36 ± 2.07) (Table 3).

The endodontic procedure difficulty score was significantly associated with the use of AAE endodontic case assessment form (*p*=0.018), where it was observed that those who used the form had a significantly lower score compared to those who did not (29.77 ± 7.71 vs. 32.52 + 8.34) (Table 4).

Table 5 shows a significant association between the use of the AAE difficulty assessment form and the overall difficulty encountered while performing root canal treatment.

The most difficult step in diagnosis was identified by 26 (14%) respondents as performing electric pulp test (ETP). This was followed by analyzing diagnostic periapical radiograph by 13 (7%) respondents and establishing pulpal diagnosis by 9 (5%) as shown in Table 6. The steps identified by the lowest number of respondents as being difficult included performing probing, gathering information about history of chief complaint, performing cold test, and performing palpation test (2% for each).

Among the three local anesthesia administration techniques, intrapulpal injection was the most difficult technique as 24% considered it as difficult and very difficult by 14%. On the other hand, 30% of the participants considered regional block as slightly difficult. Tooth build up after caries excavation was the most difficult step in isolation as 28% found it difficult and another 6% reported it as a very difficult step (Table 7).

Canal obturation was the most difficult step while performing the endodontic procedure, and 23% reported it as difficult, while 5% reported it as very difficult (Table 8).

**Figure 2:**
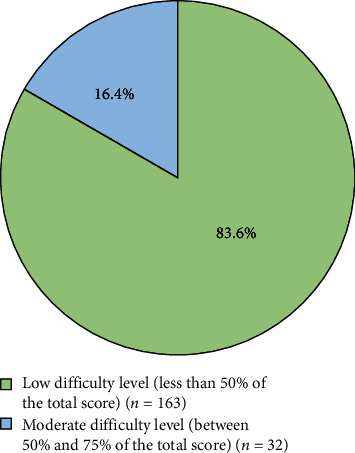
Root canal treatment overall difficulty levels.

Moderate overall difficulty of root canal treatment was reported by 16% of the participants (Figure 2).The summary of difficulty level reported by the dental students for each step when performing root canal treatment is combined in Table 9. For the diagnosis step, most of the participants reported a low difficulty level (94.4%), while 5.6% experienced a moderate difficulty level. There were 65.1% of the participants who scored <50% of the total score (low difficulty) in local anesthesia administration, and only 2.1% were classified as high difficulty in the same step. The isolation step showed a low difficulty level for 59.5% of the respondents, while only 1% experienced a high difficulty. The endodontic procedure steps difficulty level had a low difficulty level for 68.2% of the participants, while 0.5% experienced a high difficulty level.

## 4. Discussion

The study found that factors such as the students' academic level, their use of the American Association of Endodontics (AAE) endodontic case assessment form, and their academic year significantly influence these challenges. The implications of these findings can further aid in refining the design of preclinical training and clinical endodontic courses.

The results show that the overall mean difficulty score of the root canal treatment procedure was lower for 5th-year students than for 4th-year students across both universities, though the difference was not significant. However, Alrahabi [3] reported that 4th-year students were significantly more confident than 5th-year students in most of the root canal treatment steps. The difference in the results could be explained by the type of cases assigned to each group. Cases that were treated by the students in the 5th year in Alrahabi [3] were more complex and challenging than the cases treated by the 4th year. The difference between this study's results and Alrahabi [3] is that the cases that are referred to the dental students in both KSU and KSAUHS are screened in advance. In 2023, Almutairi et al. [10] concluded that the academic level did not significantly affect the student confidence while performing root canal treatment except in taking x-rays in mesial or distal shift.

According to the results of this study, the difficulty score for diagnosis was significantly associated with two main factors: the students' academic level and the use of the AAE endodontic case difficulty assessment form. Fourth year students at KSAU had shown a significantly higher diagnosis difficulty score than 5th-year students. This could suggest that the progression in academic levels has a positive effect on decreasing the perceived difficulty of diagnosis.

AAE case assessment form, provided by the American Association of Endodontists (AAE), is a tool designed to guide clinicians, faculty members, and students in assessing the complexity of any endodontic case and help in their decision-making processes [9]. In this study, students who used this form had less difficulty with not only for diagnosis but also for isolation and the overall endodontic procedure. This indicates that this tool might assist in clarifying the complexity of cases and promoting a better understanding of the procedure. Hence, the students may avoid treating complex cases and refer the case to an endodontist. This study is the first to highlight the beneficial impact of using this form for case difficulty evaluation and self-assessment among undergraduate dental students during clinical sessions. This finding corresponds to a study conducted at the Netherlands where 90% of the participants stated that the Endodontic Treatment Classification (ETC) and Dutch Endodontic Treatment Index (DETI) forms are valuable tools and guide for assessing the difficulty of root canal treatment, and thus, less difficulty was encountered during the endodontic procedure [6].

In relation to the various steps involved in root canal treatment, from access cavity preparation to temporization, the current study found that obturation was the most challenging for both 4th- and 5th-year students. This contrasts with findings from a study by Javed et al. [11] where the difficulty of obturation significantly varied with the academic level of students. Our study did not show this pattern [11].

In the current study, tooth isolation was identified as the procedure with the lowest difficulty score, mirroring findings from both Javed et al.'s [11] study and Murray and Chandler [12] research conducted in New Zealand. This lower difficulty level can be attributed to the comprehensive rubber dam isolation training sessions included in other specialties and not exclusive to endodontics. The use of the AAE endodontic case difficulty assessment form was found to be significantly related to the ease of rubber dam isolation. This suggests that the form could be an effective tool in helping students understand and manage the complexity of different steps in endodontic procedures.

The results of this study have shown a positive association between the use of the AAE case difficulty assessment form and the students' confidence while performing the root canal treatment. Also, it was previously reported that the use of the AAE form reduced the number of endodontic mishaps as well as number of visits required to complete the treatment among dental students [13].

It is generally expected that the standard of clinical teaching students receive will directly influence their competence. However, a student's perception of their competence does not always align with their actual skill level. Furthermore, the total number of clinical endodontic cases a student has performed does not necessarily reflect their competence. Yet, it is probable that with more structured clinical experience, students' confidence and competence will improve. To facilitate this, exposure to more complex endodontic cases and an increase in the number of cases treated are recommended. Additionally, extending the credit hours for the endodontic course could enable students to better perform root canal treatments. By recognizing and referring cases beyond their expertise to postgraduate endodontics residents or endodontists, students can ensure appropriate patient care while also understanding their own professional limitations. This exposure and experience will allow students to improve their skills and better handle the cases suitable for their level of competence. The AAE difficulty assessment form has shown to be a useful tool to help in assessing difficult cases thus refer the case when needed. Dental students should be familiar with such a tool and be able to use it and apply the recommendation during the clinical endodontic courses. This will help in graduating dental students who are confident to perform root canal therapy on cases that coincide with their clinical skills.

The cross-sectional study design of the current study limits the generalization of the results as it was conducted in two dental schools in Saudi Arabia, only. Another limitation is the response rate which could not be controlled since it was not a mandatory questionnaire, so the students had the right to abstain from participation which affected the response rate. This factor can be overcome by integrating it into the curriculum in future studies.

Assessment of the major difficulties encountered by dental students during endodontic treatment may assist the development of teaching methods during preclinical and clinical teaching [14, 15]. Thus, this study sets a foundation of a starting point for educators in dentistry for future studies. A prospective randomized study design to establish the causality and treatment outcome can better assess the effect of using the AAE form from several perspectives such as quality of treatment provided to the patients, students' confidence perception while providing the treatment, and a reliable feedback tool for educators.

## 5. Conclusion

The AAE difficulty assessment form was found to be useful for dental students, which enables them to differentiate between complicated and uncomplicated cases, thus lowering the difficulties encountered while performing root canal treatment. This can guide them in decision-making as a general dentist whether to perform root canal treatment on cases or it should be referred to an endodontist. The students' academic level and their practical clinical experience are factors that significantly influence the level of difficulty encountered by dental students while performing each step of root canal treatment. Since the assessment form significantly decreases the level of difficulty during root canal treatment, integrating the case assessment form into the clinical training of the dental students as requirement should be considered by dental educators. Both quantitative and qualitative long-term randomized study designs to establish the causality and treatment outcome will better assess the effect of using different case assessment forms as a feedback tool for educators to modify the learning journey of the dental students to enhance students' confidence while providing an adequate root canal therapy to their patients.

## Figures and Tables

**Table 1:** Sociodemographic and academic profile of the participants (*n* = 195).

Demographical characteristics	*n*	%
Gender		
Male	94	48.2
Female	101	51.8
University		
King Saud bin Abdulaziz University for Health Sciences (KSAU)	139	71.3
King Saud University (KSU)	56	28.7
KSAU students: your level in academic year 2021−2022 (*n* = 139)		
Year 4	62	44.6
Year 5	77	55.4
KSU students: your level in academic year 2021−2022 (*n* = 56)		
Year 4	24	42.9
Year 5	32	57.1

**Table 2:** Factors associated with diagnosis difficulty.

Factor	Diagnosis difficulty score		*P*-value
	Mean	SD	
Gender			0.12
Male	18.43	4.60	
Female	19.52	5.28	
University			0.09
King Saud bin Abdulaziz University for Health Sciences (KSAU)	18.61	4.70	
King Saud University (KSU)	19.95	5.55	
Academic level of KSAU students			0.012^*∗*^
Year 4	19.73	4.71	
Year 5	17.71	4.53	
Academic year of KSU students			0.46
Year 4	20.58	5.20	
Year 5	19.47	5.83	
Do you use the American Association of Endodontists (AAE) endodontic case assessment form?			0.005^*∗*^
Yes	18.10	4.83	
No	20.10	4.97	

^*∗*^Significant at level 0.05. SD, standard deviation.

**Table 3:** Factors associated with isolation difficulty.

Factor	Isolation difficulty score		*P*-value
	Mean	SD	
Gender			0.35
Male	6.84	2.17	
Female	7.13	2.09	
University			0.79
King Saud bin Abdulaziz University for Health Sciences (KSAU)	6.96	2.15	
King Saud University (KSU)	7.05	2.08	
Academic level of KSAU students			0.38
Year 4	7.15	2.10	
Year 5	6.82	2.20	
Academic year of KSU students			0.67
Year 4	6.92	1.91	
Year 5	7.16	2.22	
Do you use the American Association of Endodontists (AAE) endodontic case assessment form?			0.03^*∗*^
Yes	6.69	2.13	
No	7.36	2.07	

^*∗*^Significant at level 0.05. SD, standard deviation.

**Table 4:** Factors associated with endodontic procedure difficulty.

Factor	Endodontic procedure difficulty score		*P*-value
	Mean	SD	
Gender			0.40
Male	30.49	8.25	
Female	31.47	7.96	
University			0.21
King Saud bin Abdulaziz University for Health Sciences (KSAU)	30.53	8.16	
King Saud University (KSU)	32.14	7.87	
Academic level of KSAU students			0.57
Year 4	30.97	7.93	
Year 5	30.18	8.38	
Academic year of KSU students			0.28
Year 4	33.46	6.30	
Year 5	31.16	8.84	

SD, standard deviation.

**Table 5:** Factors associated with overall difficulty while performing root canal treatment.

Factor	Root canal treatment overall difficulty score		*P*-value
	Mean	SD	
Gender			0.22
Male	62.6	13.74	
Female	65.03	14.00	
University			0.40
King Saud bin Abdulaziz University for Health Sciences (KSAU)	63.32	13.69	
King Saud University (KSU)	65.18	14.42	
Academic level of KSAU students			0.13
Year 4	65.31	13.17	
Year 5	61.73	13.98	
Academic year of KSU students			0.42
Year 4	67.00	11.73	
Year 5	63.81	16.19	
Do you use the American Association of Endodontists (AAE) endodontic case assessment form?			0.005^*∗*^
Yes	61.37	14.02	
No	66.94	13.18	

^*∗*^Significant at level 0.05. SD, standard deviation.

**Table 6:** Root canal treatment step difficulty assessment (*n* = 195).

Diagnosis difficulty assessment	Very easy		Easy		Slightly difficult		Difficult		Very difficult	
1. Gathering information about the history of chief complaint	115	59%	54	28%	22	11%	4	2%	—	—
2. Performing cold test	104	53%	56	29%	31	16%	4	2%	—	—
3. Performing electric pulp test (EPT)	54	28%	54	28%	61	31%	21	11%	5	3%
4. Performing percussion test	152	78%	35	18%	8	4%	—	—	—	—
5. Performing palpation test	148	76%	36	19%	7	4%	4	2%	—	—
6. Performing bite test	69	35%	47	24%	72	37%	7	4%	—	—
7. Performing probing	132	68%	52	27%	8	4%	3	2%	—	—
8. Establishing pulpal diagnosis	87	45%	66	34%	33	17%	9	5%	—	—
9. Establishing periapical diagnosis	101	52%	59	30%	27	14%	7	4%	1	1%
10. Analyzing diagnostic periapical radiograph	65	33%	66	34%	51	26%	12	6%	1	1%
11. Analyzing diagnostic bitewing	93	48%	57	29%	40	21%	4	2%	1	1%

**Table 7:** Root canal treatment steps difficulty assessment (*n* = 195).

Local anesthesia administration assessment	Very easy		Easy		Slightly difficult		Difficult		Very difficult	
1. Performing local infiltration	148	76%	35	18%	9	5%	2	1%	1	1%
2. Performing regional block	37	19%	62	32%	58	30%	32	16%	6	3%
3. Performing intrapulpal injection	31	16%	34	17%	56	29%	47	24%	27	14%

Isolation assessment										

1. Clamp selection	51	26%	67	34%	60	31%	16	8%	1	1%
2. Rubber dam application	78	40%	82	42%	24	12%	9	5%	2	1%
3. Tooth build up after caries excavation	25	13%	43	22%	61	31%	55	28%	11	6%

**Table 8:** Root canal treatment steps difficulty assessment (*n* = 195).

Steps of nonsurgical root canal treatment	Very easy		Easy		Slightly difficult		Difficult		Very difficult	
1. Access cavity preparation	22	11%	45	23%	92	47%	30	15%	6	3%
2. Achieving straight line access	19	10%	63	32%	79	41%	32	16%	2	1%
3. Achieving glide path	23	12%	68	35%	71	36%	30	15%	3	2%
4. Using electronic apex locator to determine working length	52	27%	63	32%	57	29%	10	5%	13	7%
5. Recapitulation	78	40%	73	37%	32	16%	10	5%	2	1%
6. The use of sodium hypochlorite irrigant	116	60%	53	27%	23	12%	3	2%	0	0%
7. Master apical file selection	89	46%	62	32%	34	17%	9	5%	1	1%
8. Step back	76	39%	64	33%	42	22%	12	6%	1	1%
9. Placement of intracanal medicament	84	43%	57	29%	43	22%	10	5%	1	1%
10. Removal of intracanal medicament	68	35%	62	32%	49	25%	14	7%	2	1%
11. Master cone fit	46	24%	48	25%	74	38%	21	11%	6	3%
12. Obturation	16	8%	51	26%	74	38%	44	23%	10	5%
13. Temporization	107	55%	63	32%	21	11%	3	2%	1	1%
14. Taking diagnostic periapical radiographs such as initial working length radiograph, master file, and master cone selection radiographs during and after the treatment	44	23%	59	30%	61	31%	21	11%	10	5%

**Table 9:** Root canal treatment step difficulty levels (*n* = 195).

Question	*n*	%
Diagnosis difficulty level		
Low difficulty level (less than 50% of the total score)	184	94.4
Moderate difficulty level (between 50% and 75% of the total score)	11	5.6
Local anesthesia administration difficulty level		
Low difficulty level (less than 50% of the total score)	127	65.1
Moderate difficulty level (between 50% and 75% of the total score)	64	32.8
High difficulty level (higher than 75% of the total score)	4	2.1
Isolation difficulty level		
Low difficulty level (less than 50% of the total score)	116	59.5
Moderate difficulty level (between 50% and 75% of the total score)	77	39.5
High difficulty level (higher than 75% of the total score)	2	1
Endodontic procedure difficulty level		
Low difficulty level (less than 50% of the total score)	133	68.20
Moderate difficulty level (between 50% and 75% of the total score)	61	31.30
High difficulty level (higher than 75% of the total score)	1	0.50

## Data Availability

The data can be made available on request from the corresponding author.

## Supplementary Materials

Supplementary Materials Questionnaire S1: questionnaire for assessing dental students' perception of the utilization of a case difficulty assessment form during various stages of root canal treatment.

## References

[1] Pesonen R., Tanner T., Käkilehto T., Oikarinen-Juusola K., Laitala M.-L., Anttonen V. (2021). Usefulness of an endodontic case difficulty assessment form of root canal treatments in dental education in Finland. *Dentistry Journal*.

[2] Lynch C. D., Burke F. M. (2006). Quality of root canal fillings performed by undergraduate dental students on single-rooted teeth. *European Journal of Dental Education*.

[3] Alrahabi M. (2017). The confidence of undergraduate dental students in Saudi Arabia in performing endodontic treatment. *European Journal of Dentistry*.

[4] Davey J., Bryant S. T., Dummer P. M. H. (2015). The confidence of undergraduate dental students when performing root canal treatment and their perception of the quality of endodontic education. *European Journal of Dental Education*.

[5] Rosenberg R. J., Goodis H. E. (1992). Endodontic case selection: To treat or to refer. *The Journal of the American Dental Association*.

[6] Ree M. H., Timmerman M. F., Wesselink P. R. (2003). An evaluation of the usefulness of two endodontic case assessment forms by general dentists. *International Endodontic Journal*.

[7] Mallishery S., Chhatpar P., Banga K. S., Shah T., Gupta P. (2020). The precision of case difficulty and referral decisions: an innovative automated approach. *Clinical Oral Investigations*.

[8] Swartz D. B., Skidmore A. E., Griffin J. A. (1983). Twenty years of endodontic success and failure. *Journal of Endodontics*.

[9] Endodontic Case Difficulty Assessment Form, American Association of Endodontists (2022). https://www.aae.org/specialty/wp-content/uploads/sites/2/2022/01/CaseDifficultyAssessmentFormFINAL2022.pdf.

[10] Almutairi M., Alattas M. H., Alamoudi A. (2023). Challenges assessment in endodontics among undergraduate students. *Cureus*.

[11] Javed M. Q., Khan A. M., Bhatti U. A. (2021). Evaluation of undergraduate dental students self-perceived confidence level regarding endodontic procedures: a questionnaire survey. *Saudi Endodontic Journal*.

[12] Murray C. M., Chandler N. P. (2014). Undergraduate endodontic teaching in New Zealand: students’ experience, perceptions and self-confidence levels. *Australian Endodontic Journal*.

[13] Almohaimede A. A., AlShehri B. M., Alaiban A. A., AlDakhil R. A. (2022). Significance of endodontic case difficulty assessment: a retrospective study. *International Dental Journal*.

[14] Kaplan T., Sezgin G. P., Sönmez-Kaplan S. (2020). Dental students’ perception of difficulties concerning root canal therapy: a survey study. *Saudi Endodontic Journal*.

[15] Tavares L. G., Lima S. M. F., Lima M. G., Arruda M. P., Menegazzi T. C., Rezende T. M. B. (2019). Undergraduate dentistry students’ perception of difficulties regarding endodontic treatment. *Australian Endodontic Journal*.

