# Supplementary material for: Cross-Sectional Survey to Assess Dental Students' Perception of the Utilization of a Case Difficulty Assessment Form during Various Stages of Root Canal Therapy
Source: Int J Dent. 2024 May 29;2024:1217448. doi: 10.1155/2024/1217448 (PMC11221960; doi:10.1155/2024/1217448)
Supplement: Supplementary Materials — Questionnaire S1: questionnaire for assessing dental students' perception of the utilization of a case difficulty assessment form during various stages of root canal treatment. [file 1217448.f1.docx]

**Questionnaire**

The questionnaire includes 8 sections which are:

1. Informed Consent
2. Demographic Data
3. The use of AAE Endodontic Case Assessment Form
4. Root Canal Treatment Steps
5. Diagnosis
6. Local anesthesia administration
7. Isolation
8. Endodontic Procedure

**Section 1: Informed Consent**

1- Do you agree to participate in this study?

1. Yes
2. No

**Section 2: Demographic Data**

1. Gender:
2. Male
3. Female
4. University:
5. King Saud bin Abdulaziz University forHealth Sciences (KSAU)
6. King Saud University (KSU)
7. For KSAU students: Your level in academic year 2021-2022:
8. D4
9. D3
10. For KSU students: Your level in academic year 2021-2022:
11. 4th year
12. 5th year

**Section 3: The use of AAE Endodontic Case Assessment Form**

1. Do you use the AAE Endodontic Case Assessment Form?
2. Yes
3. No
4. Never heard about it

**Section 4: Root Canal Treatment Steps ( Difficulty Level Scale )**

The participant will answer the questions with the difficulty level of each step of root canal treatment by using a scale from 1 to 5:

1 = Very easy

2 = Easy

3 = Moderate

4 = Difficult

5 = Very difficult

**Section A: Diagnosis:**

1. Gathering information about the history of chief complaint

1 = Very easy

2 = Easy

3 = Moderate

4 = Difficult

5 = Very difficult

1. Performing cold test:

1 = Very easy

2 = Easy

3 = Moderate

4 = Difficult

5 = Very difficult

1. Performing Electric Pulp Test (EPT):

1 = Very easy

2 = Easy

3 = Moderate

4 = Difficult

5 = Very difficult

1. Performing percussion test:

1 = Very easy

2 = Easy

3 = Moderate

4 = Difficult

5 = Very difficult

1. Performing palpation test:

1 = Very easy

2 = Easy

3 = Moderate

4 = Difficult

5 = Very difficult

1. Performing bite test:

1 = Very easy

2 = Easy

3 = Moderate

4 = Difficult

5 = Very difficult

1. Performing probing:

1 = Very easy

2 = Easy

3 = Moderate

4 = Difficult

5 = Very difficult

1. Establishing pulpal diagnosis:

1 = Very easy

2 = Easy

3 = Moderate

4 = Difficult

5 = Very difficult

1. Establishing periapical diagnosis:

1 = Very easy

2 = Easy

3 = Moderate

4 = Difficult

5 = Very difficult

1. Analyzing diagnostic periapical radiograph:

1 = Very easy

2 = Easy

3 = Moderate

4 = Difficult

5 = Very difficult

1. Analyzing diagnostic bitewing:

1 = Very easy

2 = Easy

3 = Moderate

4 = Difficult

5 = Very difficult

**Section B: Local anesthesia administration:**

1. Performing local infiltration

1 = Very easy

2 = Easy

3 = Moderate

4 = Difficult

5 = Very difficult

1. Performing regional block:

1 = Very easy

2 = Easy

3 = Moderate

4 = Difficult

5 = Very difficult

1. Performing intrapulpal injection:

1 = Very easy

2 = Easy

3 = Moderate

4 = Difficult

5 = Very difficult

**Section C: Isolation:**

1. Clamp selection:

1 = Very easy

2 = Easy

3 = Moderate

4 = Difficult

5 = Very difficult

1. Rubber dam application:

1 = Very easy

2 = Easy

3 = Moderate

4 = Difficult

5 = Very difficult

1. Tooth build up after caries excavation:

1 = Very easy

2 = Easy

3 = Moderate

4 = Difficult

5 = Very difficult

**Section D: Endodontic Procedure:**

1. Access cavity preparation:

1 = Very easy

2 = Easy

3 = Moderate

4 = Difficult

5 = Very difficult

1. Achieving straight line access:

1 = Very easy

2 = Easy

3 = Moderate

4 = Difficult

5 = Very difficult

1. Achieving glide path:

1 = Very easy

2 = Easy

3 = Moderate

4 = Difficult

5 = Very difficult

1. Using electronic apex locator to determine working length:

1 = Very easy

2 = Easy

3 = Moderate

4 = Difficult

5 = Very difficult

1. Recapitulation:

1 = Very easy

2 = Easy

3 = Moderate

4 = Difficult

5 = Very difficult

1. The use of sodium hypochlorite irrigant:

1 = Very easy

2 = Easy

3 = Moderate

4 = Difficult

5 = Very difficult

1. Master apical file selection:

1 = Very easy

2 = Easy

3 = Moderate

4 = Difficult

5 = Very difficult

1. Step back:

1 = Very easy

2 = Easy

3 = Moderate

4 = Difficult

5 = Very difficult

1. Placement of intra-canal medicament:

1 = Very easy

2 = Easy

3 = Moderate

4 = Difficult

5 = Very difficult

1. Removal of intra-canal medicament:

1 = Very easy

2 = Easy

3 = Moderate

4 = Difficult

5 = Very difficult

1. Master cone fit:

1 = Very easy

2 = Easy

3 = Moderate

4 = Difficult

5 = Very difficult

1. Obturation:

1 = Very easy

2 = Easy

3 = Moderate

4 = Difficult

5 = Very difficult

1. Temporization:

1 = Very easy

2 = Easy

3 = Moderate

4 = Difficult

5 = Very difficult

1. Taking diagnostic Periapical radiographs such as initial working length radiograph, master file and master cone selections radiographs during and after the treatment:

1 = Very easy

2 = Easy

3 = Moderate

4 = Difficult

5 = Very difficult
